# Nebulized Polygonum cuspidatum-derived nanovesicles loaded with dexamethasone synergistically ameliorate acute lung injury via metabolic-immune reprogramming

**DOI:** 10.3389/fimmu.2026.1872209

**Published:** 2026-09-22

**Authors:** Zibin Jiang, Fan Shen, Fang Cheng, Jianwei Dai, Dongxing Zhao

**Affiliations:** 1 State Key Lab of Respiratory Disease, Guangzhou Medical University, Guangzhou, China; 2 School of Pharmaceutical Sciences, Sun Yat-sen University, Shenzhen, China; 3 Guangzhou Dublin International College of Life Sciences and Technology, South China Agricultural University, Guangzhou, China; 4 GMU-GIBH Joint School of Life Sciences, The Guangdong-Hong Kong-Macau Joint Laboratory for Cell Fate Regulation and Diseases, Guangzhou Medical University, Guangzhou, China; 5 National Clinical Research Center for Respiratory Disease, National Center for Respiratory Medicine, Joint International Research Laboratory of Respiratory Health, Guangdong Basic Research Center of Excellence for Respiratory Medicine, Sleep Medicine Center, Guangzhou Institute of Respiratory Health, the First Affiliated Hospital of Guangzhou Medical University, Guangzhou, China; 6 China-Portugal Artificial Intelligence and Public Health Technologies Joint Laboratory, Guangdong-Hong Kong-Macao Joint Laboratory of Respiratory Infectious Diseases, Guangdong Provincial Key Laboratory of Respiratory Disease Research, Guangzhou Medical University, Guangzhou, China

**Keywords:** acute lung injury (ALI), dexamethasone, macrophage polarization, nebulized inhalation, Polygonum cuspidatum-Derived Nanovesicles (PcDNs)

## Abstract

**Background:**

Systemic glucocorticoid therapy for acute lung injury (ALI) is frequently limited by poor pulmonary targeting and severe systemic side effects. To address these challenges, we developed a bio-inspired, inhalable nanoplatform using naturally derived nanovesicles from the medicinal plant Polygonum cuspidatum (PcDNs) to actively deliver dexamethasone (PcDNs@Dex) via nebulization.

**Methods:**

We assessed the post-nebulization stability of the nanosystem and evaluated its therapeutic efficacy in vitro and in vivo. In vitro, we investigated macrophage polarization (M1/M2) and the associated signaling pathways. Untargeted metabolomics was employed to analyze metabolic reprogramming. In vivo, a murine lipopolysaccharide (LPS)-induced ALI model was utilized to assess pulmonary targeting, alveolar damage, vascular permeability, and cytokine production.

**Results:**

Unlike conventional inert synthetic carriers, PcDNs function as intrinsic synergistic therapeutics. PcDNs@Dex maintained robust structural and functional stability post-nebulization, ensuring efficient targeted pulmonary delivery. In vitro, PcDNs@Dex exerted superior anti-inflammatory and cytoprotective effects by actively reprogramming macrophages from a pro-inflammatory M1 to a pro-resolving M2 phenotype, synergistically mediated through the coordinated inhibition of NF-κB and JAK-STAT signaling pathways. Crucially, untargeted metabolomics unveiled a novel immunometabolic mechanism: PcDNs inherently act as bioactive modulators that restore glycerophospholipid metabolic homeostasis, thereby stabilizing cell membranes and restricting pro-inflammatory mediator production at the metabolic level. In an LPS-induced ALI model, nebulized PcDNs@Dex precisely targeted the pulmonary niche, significantly attenuating alveolar damage, reducing vascular permeability, and suppressing the local cytokine storm.

**Conclusion:**

This dual intervention effectively promoted a reparative immune microenvironment while exhibiting excellent systemic safety. These results establish PcDNs@Dex as a promising, multi-target strategy that integrates targeted delivery with synergistic bioactivity for managing inflammatory lung diseases.

## 1 Introduction

Acute lung injury (ALI) and its severe form, acute respiratory distress syndrome (ARDS), represent a spectrum of devastating clinical syndromes characterized by refractory hypoxemia and non-cardiogenic pulmonary edema (1, 2). Despite the refinement of lung-protective ventilation and supportive care, ALI/ARDS continues to impose a massive global health burden, with mortality rates persistently high at 30%–40% (3–5). The pathophysiological core of ALI is the profound disruption of the alveolar-capillary barrier, initiated by the programmed death—including apoptosis and necrosis—of alveolar epithelial and vascular endothelial cells (6, 7). This barrier failure triggers an influx of protein-rich edema fluid and a self-amplifying “cytokine storm” and oxidative stress (8–10). Crucially, the progression of this pathological cascade is primarily orchestrated by the severe dysregulation of the local pulmonary immune microenvironment, where the persistent hyperactivation of M1-like pro-inflammatory alveolar macrophages relentlessly exacerbates lung parenchymal damage (5, 11).

Despite decades of investigation, a critical gap remains in the pharmacological management of ALI (12). Corticosteroids, such as dexamethasone (Dex), remain the cornerstone of anti-inflammatory therapy. However, they are plagued by a profound efficacy-toxicity dilemma. Systemic administration often fails to achieve sufficient therapeutic concentrations within the inflamed distal airspaces, while prolonged, high-dose usage frequently induces severe systemic immunosuppression, metabolic derangements, and secondary life-threatening infections (13, 14). Furthermore, most conventional small-molecule drugs target singular signaling nodes, making them inadequate against the complex, highly redundant metabolic and immunological networks driving ALI (15, 16). Consequently, there is an unmet clinical need to develop a lung-targeted, multi-functional delivery platform capable of simultaneously restoring the alveolar barrier, dampening systemic toxicity, and reprogramming the dysregulated immune microenvironment.

Plant-derived exosome-like nanovesicles (PELNVs) have recently emerged as a highly promising frontier in biomimetic nanotechnology. They overcome the inherent limitations of conventional synthetic carriers by offering superior biocompatibility, minimal immunogenicity, and intrinsic biological activities (17, 18). Unlike mammalian cell-derived exosomes, PELNVs can be mass-produced sustainably and naturally harbor a unique cocktail of parent plant-derived lipids, proteins, and active metabolites that exert synergistic therapeutic effects (19, 20). *Polygonum cuspidatum(Pc)*, a well-documented medicinal herb traditionally employed to treat respiratory ailments, is rich in potent immunomodulatory and antioxidant compounds, including stilbenes (e.g., resveratrol and polydatin), flavonoids, and anthraquinones (21, 22). However, the therapeutic potential of *Polygonum cuspidatum*-derived nanovesicles (PcDNs) for resolving acute pulmonary inflammation remains entirely unexplored.

This study addresses the critical unmet need for a targeted, nebulized delivery system that simultaneously surmounts the pulmonary barrier as an effective carrier and resolves complex immune-microenvironment dysregulation as a bioactive therapeutic, while avoiding the systemic toxicity of corticosteroid therapy. We developed and evaluated a nebulized, dexamethasone-loaded Polygonum cuspidatum-derived nanovesicle system (PcDNs@Dex) for the precision management of ALI. We hypothesized that PcDNs@Dex would function as a synergistic therapeutic strategy, combining non-invasive, targeted pulmonary delivery of Dex with the inherent capacity of PcDNs to orchestrate immunometabolic reprogramming. By harnessing this multi-target synergy, we sought to establish a safe and highly efficacious strategy to attenuate cytokine storms and promote lung tissue repair in ALI.

## 2 Materials and methods

### 2.1 Cell culture

The human bronchial epithelial cell line (BEAS-2B) and the murine macrophage cell line (RAW264.7) were purchased from the American Type Culture Collection (ATCC, Manassas, VA, USA).RAW264.7 cells were maintained in Dulbecco’s Modified Eagle Medium (DMEM; Gibco, USA) supplemented with 10% (v/v) heat-inactivated fetal bovine serum (FBS; Every Green, China) and 1% (v/v) penicillin-streptomycin (Gibco, USA). BEAS-2B cells were cultured in DMEM containing 10% FBS and 1% antibiotics. Lipopolysaccharide (LPS, E. coliO55:B5) was from Sigma-Aldrich.

### 2.2 Experimental animals

The animals used in this study were C57BL/6 mice. All mice were male, aged 3–8 weeks, and housed under standard specific pathogen-free (SPF) conditions.

### 2.3 Extraction and purification of P. cuspidatum-derived nanovesicles

Fresh roots of *Polygonum cuspidatum* were washed, cut, and homogenized in pre-cooled PBS. The homogenate was sequentially centrifuged at 4°C: 500 × g for 10 min, 2,000 × g for 20 min, and 10,000 × g for 30 min to remove debris. The final supernatant was filtered through a 0.45 μm membrane and subjected to ultracentrifugation at 150,000 × g for 90 min. The pellet was resuspended in sterile PBS for further use or stored at -80°C.

### 2.4 Transmission electron microscopy

Purified *Polygonum cuspidatum*-derived extracellular vesicles were fixed with 2-4% paraformaldehyde at 4°C. A sample aliquot (10-20 µL) was applied onto a Formvar/carbon-coated copper grid and allowed to adsorb for 10-50 minutes at room temperature. The grid was then washed with phosphate-buffered saline (PBS) and negatively stained with 2% uranyl acetate. Vesicle morphology was examined using a transmission electron microscope (JEM-1400Flash, JEOL) operating at 80-100 kV.

### 2.5 Nanoparticle tracking analysis

*Polygonum cuspidatum*-derived exosome samples were thawed on ice and appropriately diluted with sterile PBS to a concentration within the optimal detection range of 10^7^–10^9^ particles/mL. Nanoparticle Tracking Analysis was performed using an NS300 instrument (Malvern Panalytical). Three videos of 30–60 seconds duration were captured per sample. The hydrodynamic diameter and particle concentration were calculated by the instrument’s software based on the analysis of Brownian motion.

### 2.6 Dynamic light scattering and Zeta potential measurements

Purified *Polygonum cuspidatum*-derived exosome samples were thawed on ice and diluted with sterile PBS to an appropriate concentration. The hydrodynamic diameter and polydispersity index (PDI) were determined by Dynamic Light Scattering (DLS) at 25°C using a 90Plus PALS instrument (Brookhaven Instruments). Measurements were performed at a scattering angle of 90° with a 658 nm laser. The Zeta potential was measured in the same instrument using the Phase Analysis Light Scattering (PALS) mode. For each sample, measurements were performed in triplicate. The Z-Average diameter and PDI are reported, and a Zeta potential absolute value greater than ±30 mV is considered indicative of good colloidal stability. A buffer-only control was included.

### 2.7 Drug loading via electroporation

Purified *Polygonum cuspidatum*-derived exosomes were mixed with ·dexamethasone at a mass ratio of 1:1 in chilled electroporation buffer. The mixture was transferred to a pre-cooled 0.2 cm cuvette and subjected to electroporation (300 V, 150 µF, 1-2 pulses). After electroporation, the sample was incubated at 37°C for 10-30 minutes to allow membrane resealing. Unencapsulated free drug was removed by ultracentrifugation at 100,000 × g for 70 min at 4°C. The pellet containing drug-loaded exosomes (PcDNs@Dex) was resuspended in PBS for further use. Using a UV spectrophotometer, measure the absorbance of the standard series at a wavelength near 242 nm with the corresponding buffer as the reference, and plot the standard curve (R^2^ > 0.99). The encapsulation efficiency (EE) was calculated using the following formula: EE (%) = (Total drug amount - Free drug amount)/Total drug amount × 100%.

### 2.8 Cell counting kit-8 assay

Cell viability was assessed using the CCK-8 assay. Human bronchial epithelial BEAS-2B cells and mouse monocyte-macrophage RAW264.7 cells were seeded into 96-well plates at a density of 5 × 10³ cells per well. After 24 hours of incubation, the culture medium was replaced with fresh medium containing various concentrations (0, 1, 5, 10, 25, 50 μg/mL) of *Polygonum cuspidatum*-derived nanovesicles (PcDNs) or dexamethasone-loaded PcDNs (PcDNs@Dex). Following a 24- or 48-hour treatment period, 10 μL of CCK-8 solution was added to each well and incubated for 2 hours. The absorbance at 450 nm (OD_450_) was measured using a microplate reader. Data are presented as mean ± SEM. Statistical analysis was performed using one-way ANOVA. The Cell counting kit-8 is produced by APE×BIOs.

### 2.9 Confocal microscopy

RAW264.7 cells were seeded on confocal dishes and incubated with DID-labeled *Polygonum cuspidatum*-derived exosomes for 12 hours. Cells were then washed, fixed with 4% paraformaldehyde, and stained with DAPI. Imaging was performed using a confocal microscope (LSM 880 NLO, ZEISS) with a 40x oil immersion objective. For excitation, a 405 nm laser was used for DAPI and a 633 nm laser for DID. Emission was collected at 410–480 nm (DAPI) and 650–720 nm (DID). Z-stack images were acquired with a step size of 0.5–1 μm.

### 2.10 ELISA

The levels of the pro-inflammatory cytokines tumor necrosis factor-alpha (TNF-α), interleukin-1 beta (IL-1β), and interleukin-6 (IL-6) were quantified. Measurements were performed in two types of samples: cell culture supernatants collected from treated RAW264.7 cells and obtained from mouse models. Elisa uses the brand MultiSciences.

### 2.11 Immunofluorescence

For immunofluorescence staining, RAW264.7 cells in the logarithmic growth phase were seeded onto sterile glass coverslips placed in 12-well plates at a density of 1-5×10^+^ cells per well in complete culture medium and incubated for 16-24 hours until 60-70% confluent. Following treatment, cells were gently washed with cold PBS, fixed with 4% paraformaldehyde for 20 minutes at room temperature, and washed again. For intracellular antigen detection (IL-6, IL-1β, CD206), cells were permeabilized with 0.1-0.3% Triton X-100 for 10 minutes; for surface antigen CD86, a lower concentration (0.05%) or omission of this step was applied. After blocking with 5% BSA for 1 hour, cells were incubated overnight at 4°C with primary antibodies diluted in 5% BSA. After washing, cells were incubated with fluorophore-conjugated secondary antibodies and DAPI for 1 hour at room temperature in the dark. Coverslips were then mounted onto slides with an anti-fade mounting medium. Imaging was performed using a fluorescence or confocal microscope with appropriate filter sets. Negative controls were included, and all steps were optimized to prevent fluorescence quenching and non-specific binding.

### 2.12 Quantitative real-time PCR

Total RNA was reverse transcribed into cDNA. qPCR was performed using a SYBR Green master mix on a LightCycler^®^ 96 system (Roche). The reaction mixture contained cDNA template, gene-specific primers (final concentration 0.3 μM), and qPCR mix. The thermal cycling conditions were: initial denaturation at 95°C for 10 min, followed by 40 cycles of 95°C for 15 sec, 60°C for 20 sec, and 72°C for 30 sec. A melt curve analysis was performed to confirm amplification specificity. The relative gene expression was calculated using the 2^(-ΔΔCt) method, with GAPDH as the endogenous control. TRIzol reagent was purchased from Takara(Japan). The EasyScript All-in-One First-Strand cDNA Synthesis SuperMix and 2x SYBR Green qPCR Mix were from TransGen Biotech (China). The primers of qPCR were list in the **Table 1**. The primers were designed using NCBI Primer-BLAST and validated via melt-curve analysis to ensure amplification specificity.

**Table 1:** Primer sequences.

Gene	Forward primer sequence5’->3’	Reverse primer sequence5’->3’
H-*Actin*	GCACCACACCTTCTACAATGAG	ATAGCACAGCCTGGATAGCAAC
H-*TNFα*	TCTTCTCCTTCCTGATCGTGG	AGGGCTGATTAGAGAGAGGTCC
H-*IL-6*	TACCCCCAGGAGAAGATTCC	TTTTCTGCCAGTGCCTCTTT
H-*IL-1β*	GAAATGATGGCTTATTACAGTGGC	TTGCTGTAGTGGTGGTCGGAG
M-*Actin*	GGCTGTATTCCCCTCCATCG	CCAGTTGGTAACAATGCCATGT
M-*TNFα*	CTGAACTTCGGGGTGATCGG	GGCTTGTCACTCGAATTTTGAGA
M-*IL-6*	TCTATACCACTTCACAAGTCGGA	GAATTGCCATTGCACAACTCTTT
M-*IL-1β*	GAAATGCCACCTTTTGACAGTG	TGGATGCTCTCATCAGGACAG
M-*iNOS*	ATCTTGGAGCGAGTTGTGGATTGTC	TAGGTGAGGGCTTGGCTGAGTG
M-*TGF-β1*	GGCTACTGCCGCTTCTGCTC	CCAGCTCCATGTCGATGGTCTTG

### 2.13 Western blotting

Total protein was extracted from cells or tissues using RIPA lysis buffer and quantified by the BCA method. Equal amounts of protein were separated by SDS-PAGE and transferred onto PVDF membranes. After blocking, the membranes were incubated overnight at 4°C with primary antibodies against key signaling molecules: p65, IκBα (NF-κB pathway), JAK2, and STAT6 (JAK-STAT pathway). Following incubation with HRP-conjugated secondary antibodies, protein bands were visualized using enhanced chemiluminescence (ECL). β-Actin was used as a loading control. Band intensities were quantified using ImageJ software. Primary antibodies against B-actin, IkBa, and STAT-6 were sourced from Abcam (UK). Antibodies against NF-kB p65 and JAK2 were also from Abcam. Horseradish peroxidase (HRP)-conjugated goat anti-mouse IgG secondary antibody was purchased from ServiceBio (China). The Bradford protein assay dye, RIPA lysis buffer, protease inhibitor cocktail and ECL enhanced chemiluminescence substrate.

### 2.14 Annexin V-FITC/PI apoptosis assay

Cell apoptosis was assessed using an Annexin V-FITC/PI apoptosis detection kit. Treated RAW264.7 cells (both adherent and floating) were collected, washed with cold PBS, and resuspended in binding buffer. The cells were stained with Annexin V-FITC and PI in the dark for 15 min at room temperature and then analyzed by flow cytometry, acquiring at least 10,000 events per sample. The percentages of viable, early apoptotic, late apoptotic, and necrotic cells were quantified using FlowJo software. Experiments were performed in triplicate. The Annexin V-FITC/PI apoptosis detection kit uses the brand of BD Biosciences.

### 2.15 Metabolomics

Untargeted metabolomic profiling was performed on RAW264.7 cells from three experimental groups: control (CTRL), lipopolysaccharide-treated (LPS), and LPS treated with *Polygonum cuspidatum*-derived nanovesicles (LPS+PcDNs), with three biological replicates per group. Cellular metabolites were extracted on ice using ice-cold 80% methanol aqueous solution containing internal standards, followed by vortexing and three freeze-thaw cycles for complete lysis. After centrifugation, the supernatant was processed and analyzed by liquid chromatography-mass spectrometry (LC-MS). Chromatographic separation was achieved on a Waters ACQUITY Premier HSS T3 column (1.8 μm, 2.1 × 100 mm) with a gradient of 0.1% formic acid in water and acetonitrile. Mass spectrometry was conducted on a Thermo Q Exactive HF-X instrument in both positive and negative electrospray ionization modes, with full MS and data-dependent MS/MS acquisition. Raw data were processed using XCMS for peak picking, alignment, and grouping. Peaks with a missing rate >50% were filtered, missing values were imputed via k-nearest neighbor algorithm, and data were normalized to total protein concentration. Metabolites were identified by matching accurate mass and MS/MS spectra against databases. Rigorous quality control was applied, requiring a coefficient of variation below 0.3 for metabolites in pooled quality control samples and demonstrating high instrument stability via total ion chromatogram overlap and Pearson correlation analysis. Unsupervised principal component analysis (PCA) and supervised orthogonal partial least squares-discriminant analysis (OPLS-DA) were performed to assess data quality, group separation, and to build predictive models. Model validity was confirmed via permutation testing. altered metabolites were screened based on a variable importance in projection (VIP) score >1.0 from the OPLS-DA model and a p-value <0.05 from univariate statistical analysis. KEGG pathway enrichment analysis was subsequently performed on these differential metabolites to identify the key metabolic pathways regulated by PcDNs intervention.

### 2.16 Hematoxylin and eosin Staining

Paraffin-embedded lung tissue sections (5 μm thick) were deparaffinized in xylene and rehydrated through a graded ethanol series. After rinsing in distilled water, sections were stained with hematoxylin for 5 minutes, differentiated in 1% hydrochloric acid ethanol, and blued in running tap water. Subsequently, sections were counterstained with eosin for 2 minutes, followed by dehydration through graded ethanols, clearing in xylene, and mounting with neutral balsam. Stained sections were examined under a light microscope for histopathological assessment. The following three parameters were assessed: (1) Number of alveoli per high-power field (HPF): Alveoli were manually counted in each 400× field using the ImageJ cell counter plugin. Only complete alveoli with identifiable alveolar walls were included. To avoid double-counting bias, partial alveoli touching the left and upper borders of the field were excluded, while those touching the right and lower borders were included, following the standard unbiased counting frame principle. (2) Alveolar septal thickness (µm): Alveolar septal thickness was measured as the average distance between adjacent alveolar walls. Using the ImageJ straight-line tool, ten random measurements perpendicular to the alveolar wall were taken per field. The mean value of these measurements was calculated for each field, and the average across five fields per animal was used for statistical analysis. (3) Mean linear intercept (MLI, µm)​: MLI was measured as an indicator of alveolar airspace enlargement. A grid of parallel test lines of known total length (L) was superimposed on each image using ImageJ. The number of intersections between the test lines and alveolar walls (I) was counted. MLI was calculated using the formula: MLI = L/I. Ten equidistant lines were applied per field, and the average MLI value was calculated from the five fields per animal.

### 2.17 Immunohistochemistry

Immunohistochemical staining for IL-6, IL-1β, and TNF-α was performed on paraffin-embedded mouse lung sections. After deparaffinization, rehydration, and antigen retrieval, endogenous peroxidase activity was blocked. Sections were incubated overnight at 4°C with respective primary antibodies, followed by incubation with a biotinylated secondary antibody and an avidin-biotin-peroxidase complex. Signal was developed using DAB, and sections were counterstained with hematoxylin. Stained sections were examined under a light microscope for cytokine expression analysis.

### 2.18 Flow cytometry

Flow cytometric analysis was performed to assess macrophage polarization and surface marker expression. For *in vitro* studies, RAW264.7 macrophages were treated as indicated, harvested, and washed with cold PBS. Cells were stained with fluorescently labeled antibodies against CD80 and CD86 (for M1 phenotype) or CD206 (for M2 phenotype) in staining buffer for 30 minutes at 4°C in the dark. After washing, cells were resuspended in PBS containing 1% BSA and analyzed immediately using a flow cytometer. For *in vivo* studies, single-cell suspensions were prepared from lung tissues by mechanical dissociation and enzymatic digestion, followed by erythrocyte lysis. Cells were then stained with antibodies against CD206 and other relevant markers to evaluate macrophage subsets. For flow cytometry, fluorescently labeled antibodies against mouse F4/80(FITC), CD11b (FITC), CD86 (PE), and CD206 (PE) were obtained from BioLegend (USA). The Annexin V-FITC/PI Apoptosis Detection Kit was from BD Biosciences (USA).

### 2.19 Statistical analysis

All data are presented as mean ± standard deviation (SD). Comparisons among multiple groups were performed using one-way analysis of variance (ANOVA) followed by Tukey’s post-hoc test. A p-value of less than 0.05 was considered statistically significant. All statistical analyses were conducted using GraphPad Prism software (Version 8.0).

## 3 Results

### 3.1 Preparation and physicochemical characterization of PcDNs and PcDNs@Dex

This study selected *Polygonum cuspidatum* as the source of extracellular vesicles, mainly based on its pharmacological basis as a traditional Chinese medicine and the alignment of its active components with the therapeutic goals for acute pneumonia. In order to obtain high-quality exosomes and verify their suitability for aerosol delivery, I first conducted a systematic characterization experiment on the extracellular vesicles derived from *Polygonum cuspidatum*. Nanoparticle tracking analysis confirmed that the main peak of the particle size distribution curve was tightly concentrated at 130 nanometers (**Figure 1A**), and the particle size distribution was in line with the conventional size distribution of exosomes. The DLS dynamic light scattering experiments before and after aerosolization of the extracellular vesicles derived from *Polygonum cuspidatum* revealed that the particle size distribution was almost overlapping, indicating good structural stability of the exosomes (**Figure 1B**). However, it was found that the potential of the extracellular vesicles derived from *Polygonum cuspidatum* changed before and after aerosolization, which might be a minor effect caused by aerosolization and whether it has an impact on the function remains to be further investigated in subsequent experiments (**Figure 1C**). Next, transmission electron microscopy was performed, and it was found that the morphology of the extracellular vesicles before and after aerosolization did not show any significant changes (**Figures 1D, E**). The encapsulation efficiency before and after aerosolization also did not show any significant changes (**Figure 1F**). Finally, the protein content before and after aerosolization under the same experimental volume conditions did not show any significant changes (**Figure 1G**). These data proved that the extracellular vesicle particle system derived from *Polygonum cuspidatum* has high homogeneity, excellent physical structure stability, and laid the foundation for aerosol delivery.

**Figure 1:**
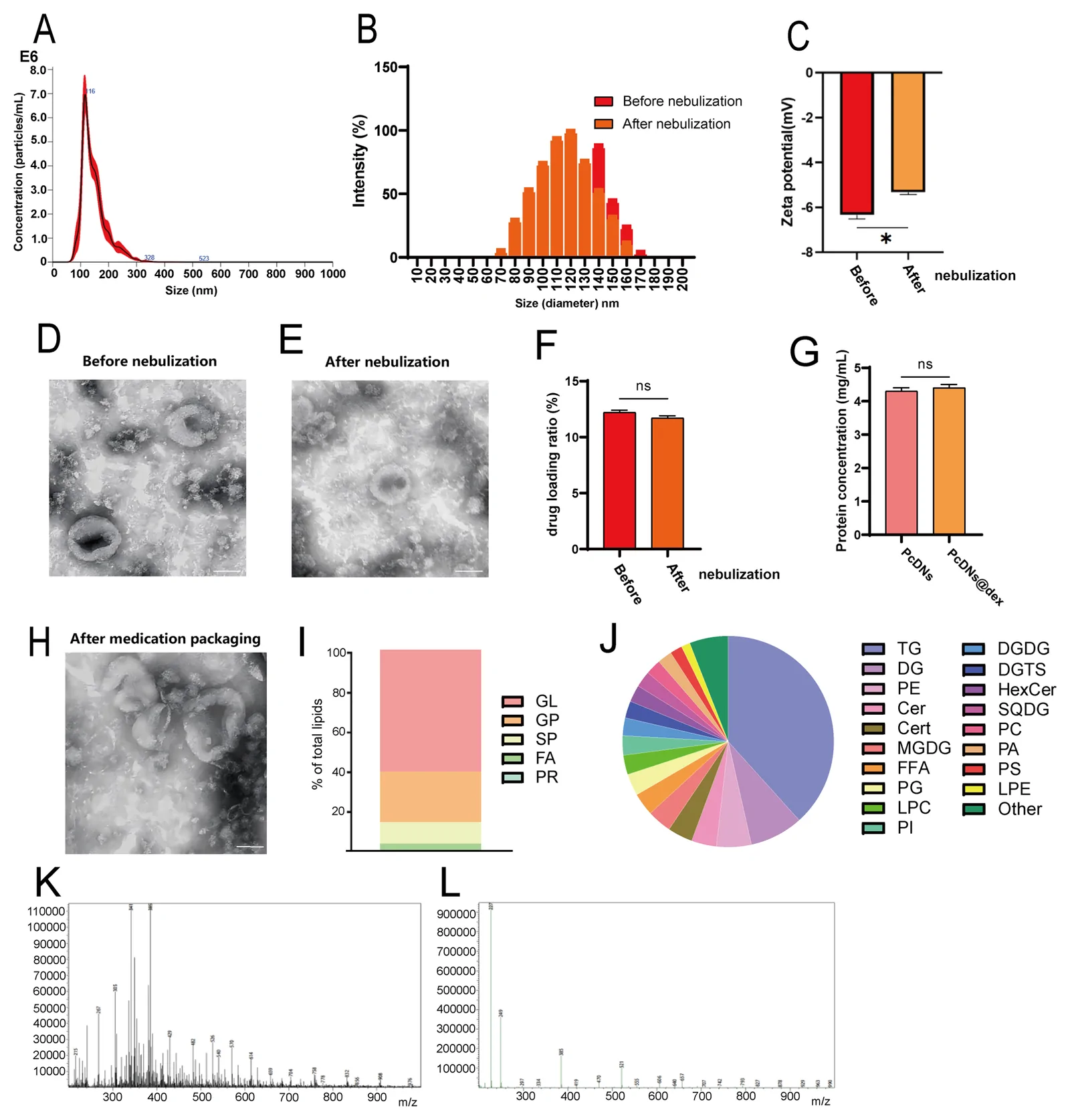
Characterization, and nebulization stability of PcDNs and PcDNs@Dex. **(A)** Size and concentration of PcDNs (n=3). **(B)** Hydrodynamic diameter before and after nebulization (n=3). **(C)** Zeta potential before and after nebulization (n=3). **(D, E)** Representative TEM images before **(D)** and after **(E)** nebulization (Scale bar = 100 nm). **(F)** Drug loading efficiency of PcDNs@Dex before and after nebulization (n=3). **(G)** Protein content before and after drug loading (n=3). **(H)** Representative TEM image of PcDNs@Dex (Scale bar = 100 nm). **(I, J)** Lipid composition analysis of PcDNs. Bar chart of major lipid classes **(I)**. Pie chart of specific lipid species **(J)**. TG, Triacylglycerol; DG, Diacylglycerol; PE, Phosphatidylethanolamine; Cer, Ceramide; CerP (formerly listed as Cert in the figure), Cerosylphospholipid (or Ceramide Phosphate, context-dependent); MGDG, Monogalactosyldiacylglycerol; FFA, Free Fatty Acid; PG, Phosphatidylglycerol; LPC, Lysophosphatidylcholine; PI, Phosphatidylinositol; DGDG, Digalactosyldiacylglycerol; DGTS, Diacylglyceryltrimethylhomoserine; HexCer, Hexosylceramide; SQDG, Sulfoquinovosyldiacylglycerol; PC, Phosphatidylcholine; PA, Phosphatidic Acid; PS, Phosphatidylserine; LPE, Lysophosphatidylethanolamine; Other, Other Lipids. **(K, L)** Mass spectrometry profile of PcDNs. *P<0.05, not significant.

Further analysis of the extracellular vesicles derived from *Polygonum cuspidatum* revealed that the transmission electron microscopy images after encapsulation showed that the vesicle structure was basically maintained (**Figure 1H**). After conducting lipidomics sequencing analysis on the extracellular vesicles derived from *Polygonum cuspidatum*, it was found that the membranes were rich in functional lipids, such as triglycerides (38.32%), diacylglycerols (8.16%), and phosphatidylglycerolamine (5.32%). These substances together formed a stable and stable membrane structure, which was crucial for maintaining their integrity (**Figures 1I, J**). Further mass spectrometry analysis also suggested that the extracellular vesicles themselves might be rich in natural anti-inflammatory compounds such as resveratrol (**Figures 1K, L**).

### 3.2 Cellular uptake, biocompatibility, and anti-inflammatory potency of PcDNs@Dex

Based on the cell experiment data, I evaluated the functional stability of extracellular vesicles (PcDNs) derived from *Polygonum cuspidatum* and their dexamethasone-loaded complexes (PcDNs@Dex) before and after nebulization. By establishing an LPS-induced RAW264.7 macrophage inflammation model and detecting the mRNA expression levels of key pro-inflammatory cytokines IL-6 and TNF-α, I found that the expression of these factors was effectively inhibited after intervention with PcDNs or PcDNs@Dex. Meanwhile, there was no significant statistical difference in the inhibitory effects on IL-6 and TNF-α between the nebulized PcDNs and PcDNs@Dex treatment groups and their corresponding pre-nebulization treatment groups (**Figures 2A–D**). This result indicates that the physical process of nebulization did not significantly weaken the *in vitro* anti-inflammatory activity of extracellular vesicles derived from *Polygonum cuspidatum* and their drug-loaded complexes, demonstrating from a functional perspective that the prepared exosome delivery system can maintain its biological activity and has good functional stability after nebulization.

**Figure 2:**
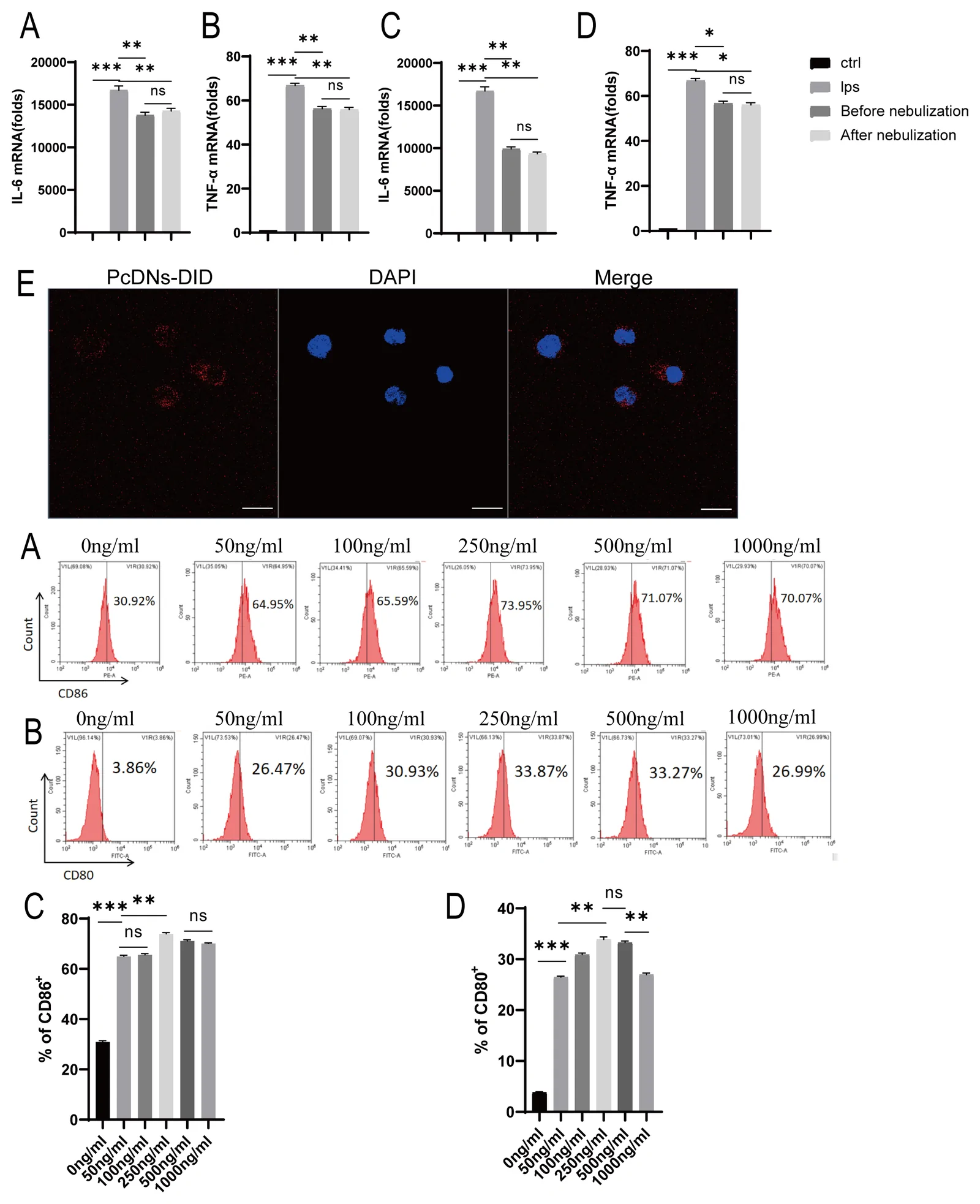
Evaluation of nebulization efficacy stability and optimization of LPS-induced inflammation model in RAW264.7 macrophages. **(A–D)** Anti-inflammatory efficacy of PcDNs **(A, B)** and PcDNs@Dex(C,D) before and after nebulization in LPS-induced RAW264.7 cells. Levels of IL-6 and TNF-α are shown. Data are presented as mean ± SD (n=3). **(E)** Cellular uptake of PcDNs in RAW264.7 cells. Representative confocal laser scanning microscopy (CLSM) images. **(F, G)** Flow cytometric analysis of CD80 and CD86 expression in RAW264.7 cells treated with increasing concentrations of LPS. Representative plots **(F)** and quantitative analysis **(G)**. **(H, I)** Quantitative analysis of CD80^+^ and CD86^+^ cell percentages. Data are presented as mean ± SD (n=3). Statistical significance was determined by one-way ANOVA with appropriate post-hoc test. *P<0.05, **P < 0.01,***P < 0.001. ns, not significant.

To exert their therapeutic effects, nanovesicles must be efficiently internalized by target cells and maintain a high safety profile. We first evaluated the cellular uptake of PcDNs in RAW264.7 macrophages. Confocal laser scanning microscopy demonstrated that fluorescently labeled PcDNs were efficiently internalized and localized within the cell lines (**Figure 2E**). This robust internalization suggests that PcDNs are highly compatible with the plasma membranes of lung-relevant cells, facilitating the intracellular delivery of encapsulated therapeutics. we established and optimized an *in vitro*model of inflammation. Dose- and time-dependent screening identified the optimal LPS stimulation conditions to robustly induce an inflammatory response in both RAW264.7 and BEAS-2B cells (**Supplementary Figure 2**). Flow cytometric analysis confirmed that LPS treatment successfully promoted M1-type macrophage polarization, as evidenced by a dose-dependent increase in the surface expression of the activation markers CD80 and CD86 (**Figures 2F, G**). In the initial model establishment phase, we only presented M1 markers (CD80/CD86) to confirm successful inflammatory induction by LPS. Considering the expression intensity of comprehensive pro-inflammatory factors and cell activity, the optimal modeling conditions for RAW264.7 cells were determined to be 50 ng/ml LPS stimulation for 12 hours, and for BEAS-2B cells, 100 ng/ml LPS stimulation for 12 hours. Under these conditions, stable inflammatory responses can be induced in both cell types, which are suitable for the subsequent evaluation of the anti-inflammatory activity of drugs (**Figures 2H, I**).

Next, we assessed the biocompatibility of the nanoplatform. The CCK-8 assay confirmed that both empty PcDNs and drug-loaded PcDNs@Dex exhibited negligible cytotoxicity in RAW264.7 macrophages and BEAS-2B bronchial epithelial cells, with cell viability remaining above 95% after 24 and 48 hours of incubation at the chosen therapeutic concentration (25 µg/mL) and showing no statistically significant difference compared to the untreated control group (**Figures 3A, B**). This high cell viability rules out the possibility that the observed reduction in IL-6 and other pro-inflammatory cytokines was due to a decrease in cell number. Therefore, the potent anti-inflammatory effect of PcDNs@Dex is attributed to active suppression of intracellular inflammatory signaling rather than nonspecific cytotoxicity.

**Figure 3:**
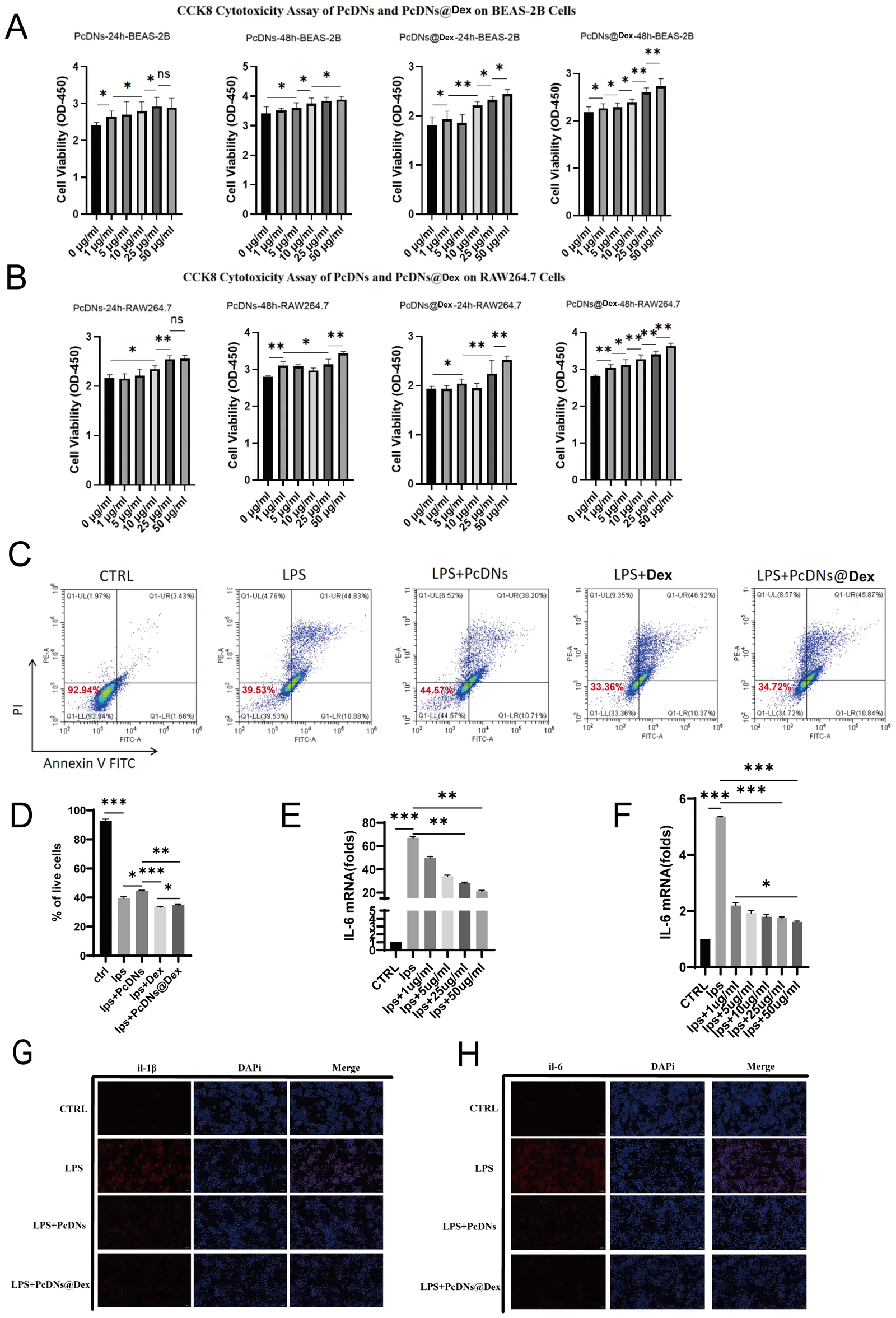
Biocompatibility, anti-apoptotic effects, and anti-inflammatory activity of PcDNs@Dex *in vitro*. **(A, B)** Cytotoxicity evaluation of PcDNs and PcDNs@Dex in BEAS-2B **(A)** and RAW264.7 **(B)** cells. Data are presented as mean ± SD (n=5). **(C, D)** Apoptosis analysis of treated cells. Representative flow cytometric plots **(C)** and quantitative data **(D)**. Data are presented as mean ± SD (n=3). **(E, F)** Optimization of therapeutic concentrations of PcDNs@Dex in BEAS-2B **(E)** and RAW264.7 **(F)** cells. Data are presented as mean ± SD (n=3). **(G, H)** Immunofluorescence staining of IL-6 **(G)** and IL-1β **(H)** in cells under different treatments. All statistical analyses were performed using one-way ANOVA with appropriate post-hoc tests. *P<0.05, **P < 0.01,***P < 0.001. ns, not significant.

These results indicate that PcDNs@Dex is a safe delivery vehicle, satisfying the stringent biosafety requirements for pulmonary administration. Beyond modulating cytokine release, we investigated the cytoprotective effect of PcDNs@Dex. LPS challenge induced apoptosis in RAW264.7 cells. Treatment with PcDNs@Dex showed a mild cytoprotective trend, as reflected by a modest reduction in the percentage of apoptotic cells (**Figures 3C, D**). However, we acknowledge that the primary therapeutic mechanism of PcDNs@Dex is its potent anti-inflammatory and immunometabolic reprogramming capacity, rather than a direct anti-apoptotic effect. Therefore, the subsequent mechanistic analyses in this study focus on its immunomodulatory and metabolic regulatory functions. Based on the data of both cell viability and anti-inflammatory potency, I ultimately chose 25 µg/ml as the uniform treatment concentration for subsequent cell experiments to achieve the optimal anti-inflammatory effect while ensuring biosafety.

Dose-response studies further determined the optimal therapeutic concentration of PcDNs@Dex for maximal anti-inflammatory effect in both cell types (**Figures 3E, F**). The potent cytokine suppression was visually confirmed at the protein level by immunofluorescence staining, which showed markedly reduced expression of IL-6 and IL-1β in cells treated with PcDNs@Dex (**Figures 3G, H**).

Collectively, these findings establish that the PcDNs@Dex platform possesses excellent biocompatibility, is efficiently internalized by target lung cells, and exerts potent anti-inflammatory, immunomodulatory, and anti-apoptotic effects *in vitro*. This provides a solid foundation for its subsequent mechanistic exploration and *in vivo* application.

### 3.3 PcDNs@Dex reprograms macrophage polarization and inhibits inflammatory signaling

To elucidate whether the anti-inflammatory effects of PcDNs@Dex were mediated through the active reprogramming of the immune microenvironment, we investigated its influence on macrophage polarization. The transition from a pro-inflammatory M1 phenotype to an anti-inflammatory M2 phenotype is a pivotal clinical indicator for the resolution of acute lung injury.

Immunofluorescence staining revealed that LPS stimulation markedly increased the expression of the M1 marker CD86, while treatment with PcDNs@Dex reduced its intensity. Conversely, the expression of the M2 marker CD206 was enhanced by PcDNs@Dex intervention, visually indicating a phenotypic shift from M1 to M2 (**Figures 4A, B**). Flow cytometric analysis quantitatively confirmed this reprogramming. LPS challenge expanded the population of CD11b^+^CD86^+^ M1-like macrophages, which was markedly attenuated by PcDNs@Dex. Concurrently, PcDNs@Dex treatment led to a substantial increase in the percentage of CD11b^+^CD206^+^ M2-like macrophages (**Figures 4C–F**). These results demonstrate that PcDNs@Dex actively facilitates a phenotypic switch that favors and immune homeostasis.

**Figure 4:**
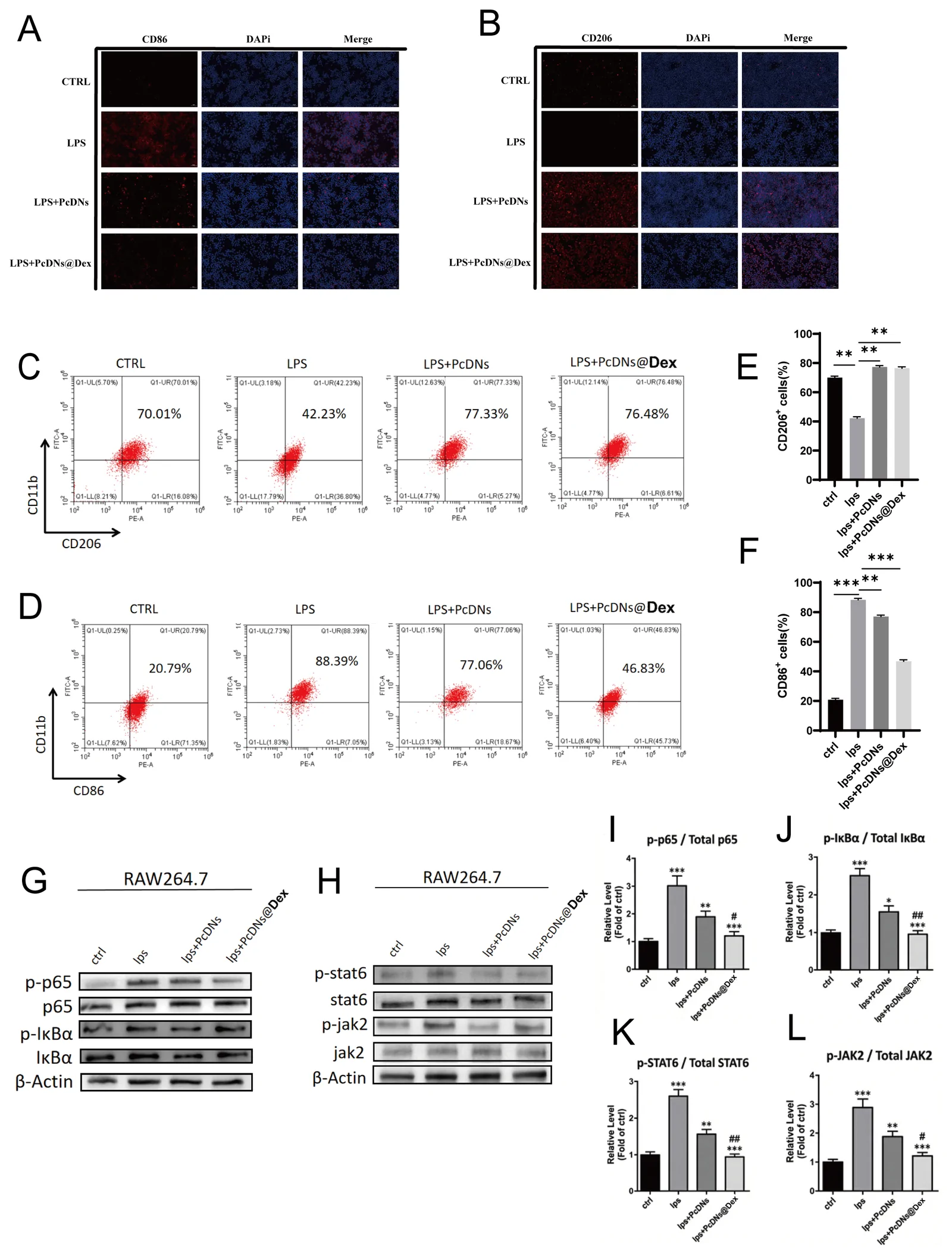
PcDNs@Dex modulates macrophage polarization and inhibits inflammatory signaling pathways.​ **(A, B)** Representative immunofluorescence images of CD86 **(A)** and CD206 **(B)** in macrophages under different treatment conditions. **(C, D)** Flow cytometric analysis of macrophage polarization. Representative scatter plots showing the percentage of CD11b^+^CD206^+^ (M2-like) cells **(C)** and CD11b^+^CD86^+^ (M1-like) cells **(D)**. **(E, F)** Quantitative analysis of CD206^+^ **(E)** and CD86^+^ **(F)** cell percentages from flow cytometry. **(G–L)** Western blot analysis of NF-κB (p-p65, p65, p-IκBα, IκBα) and JAK/STAT (p-JAK2, JAK2, p-STAT6, STAT6) pathway proteins. β-actin served as loading control. Data are presented as mean ± SD (n=3). Statistical significance was determined by one-way ANOVA followed by Tukey’s post-hoc test. *P < 0.05, **P < 0.01, ***P < 0.001, # significant difference with respect to the control with a p-value < 0.05.

To dissect the molecular basis underlying this immunomodulatory effect, we analyzed the activation of key intracellular signaling cascades, specifically the NF-κB and JAK/STAT pathways, which are major drivers of the inflammatory response. Western blot analysis demonstrated that LPS stimulation triggered the robust phosphorylation of p65 and IκBα, indicating the activation of the classical NF-κB pathway. However, treatment with PcDNs@Dex suppressed these phosphorylation events (**Figures 4G–J**). In parallel, PcDNs@Dex treatment also effectively inhibited the LPS-induced phosphorylation of JAK2 and STAT6, key components of the JAK/STAT signaling axis (**Figures 4G–J**). This synergistic inhibition of both NF-κB and JAK/STAT pathways underscores the multi-target regulatory capacity of the PcDNs@Dex platform.

Collectively, these data demonstrate that PcDNs@Dex does not merely block inflammatory output but actively re-orchestrates the macrophage landscape toward a resolving phenotype by modulating pivotal signal transduction nodes.

### 3.4 Non-targeted metabolomics reveals metabolic reprogramming by PcDNs

To gain a systemic understanding of how PcDNs modulate the internal metabolic state of macrophages, we performed a non-targeted metabolomics analysis using LC-MS/MS. A total of 1,445 metabolites were detected, of which 1,140 were identified at Level 2, providing a comprehensive snapshot of the cellular metabolic landscape (**Figure 5A**).

**Figure 5:**
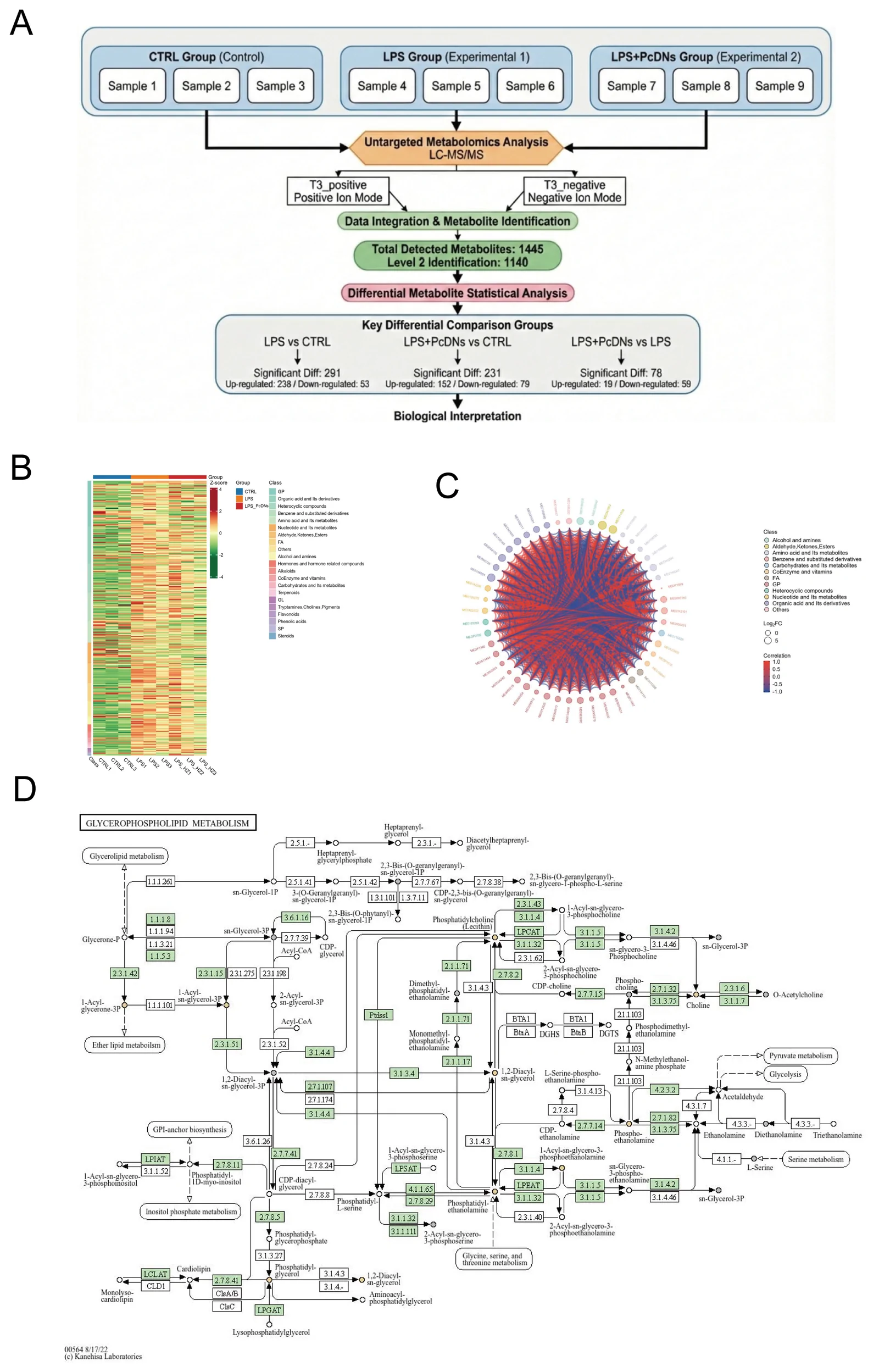
Non-targeted metabolomic analysis reveals metabolic reprogramming in macrophages regulated by PcDNs. **(A)** Experimental workflow of the metabolomic study. **(B)** Heatmap of differentially expressed metabolites. **(C)** Correlation network of key metabolites. **(D)** Glycerophospholipid metabolism pathway map.

To comprehensively assess the quality and reliability of the untargeted metabolomics data, we performed a systematic analysis of the quality control (QC) samples. The total ion chromatograms (TICs) demonstrated a high degree of overlap in both retention time and response intensity across all QC samples, indicating excellent instrument stability throughout the entire data collection process (**Supplementary Figure 3A**). The reproducibility of the data was further confirmed by Pearson correlation analysis among QC samples, with all correlation coefficients approximating 1 (**Supplementary Figure 3B**). Moreover, the response coefficients of variation (CVs) for internal standards in the QC samples were all below 0.05, attesting to the high precision of the entire workflow from sample preparation to instrumental analysis (**Supplementary Figure 3B**). Finally, evaluation of the CV distribution for all detected metabolites revealed that over 85% of metabolites had a CV value below 0.5, and more than 75% had a CV below 0.3 in the QC samples. This robustly demonstrates the high stability and reliability of the current untargeted metabolomics dataset, which meets the stringent requirements for subsequent differential metabolite screening and bioinformatic analysis (**Supplementary Figure 3C**).

During the principal component analysis (PCA) stage, an unsupervised multivariate statistical method was first applied to the metabolomics data for dimensionality reduction, aiming to assess the overall metabolic differences between groups and the within-group variability. PCA transforms potentially correlated variables into linearly uncorrelated principal components via orthogonal transformation, thereby revealing the internal data structure while retaining the original information. Specifically, the data were subjected to unit variance (UV) scaling normalization using the prcompfunction in R software, calculated as x=(x−μ)/σ to ensure the data conformed to a standard normal distribution. The overall sample PCA, which included all experimental and quality control (QC) samples, revealed a clear separation between groups in the PCA score plot. Principal component analysis (PCA) revealed clear separation among the Control, LPS, and LPS+PcDNs groups, with the first three principal components (PC1, PC2, and PC3) accounting for the major variations in the dataset. The tight clustering of quality control (QC) samples confirmed high instrument stability throughout the analytical process (**Supplementary Figure 4A**). Notably, we observed some intra-group dispersion within the LPS and LPS+PcDNs groups, such as the proximity between LPS3 and LPS_PcDNs2. This is a common phenomenon in metabolomics studies involving live cells, as the dynamic and complex nature of the inflammatory response can introduce biological variance within stimulated groups. Despite this intra-group variability, the supervised orthogonal partial least squares-discriminant analysis (OPLS-DA) model yielded high predictive validity (Q^2^ > 0.95 for both pairwise comparisons), and permutation tests confirmed that the inter-group separation is statistically robust and not attributable to overfitting (**Supplementary Figure 5**). Therefore, the observed metabolic shifts between groups are biologically meaningful and reliably reflect the intervention effects of PcDNs.

To further monitor instrument performance, univariate statistical process control based on the PCA model was applied to the QC samples, and a PC1 control chart was plotted. The detection sequence of the samples is displayed on the x-axis, and the corresponding PC1 score is shown on the y-axis. Green dots represent QC samples, and black dots represent experimental samples. The yellow and red lines define the ranges of ±2 and ±3 standard deviations, respectively. The results show that the PC1 scores of all QC samples remained within the control limits, demonstrating stable mass spectrometry signals and high data reliability (**Supplementary Figure 4B**).

Following the overall metabolomics data quality assessment via principal component analysis (PCA) and the analysis of separation trends among the three groups (CTRL, LPS, and LPS-PcDNs), to further quantify the metabolic profile differences between specific paired groups and to establish a predictive classification model, this study performed supervised Orthogonal Partial Least Squares-Discriminant Analysis (OPLS-DA) on two critical comparisons: CTRL vs. LPS and LPS vs. LPS-PcDNs. The OPLS-DA results revealed a clear separation of the two groups along the first predictive component (Tp1) in the score plot for the CTRL vs. LPS comparison. The model validation parameters, R^2^Y (the model’s explained variance for the Y-matrix) and Q^2^ (the model’s predictive capability), were as high as 0.992 and 0.985, respectively, both approaching 1. This indicates the model possesses strong explanatory power and reliable predictability. A 200-permutation test yielded a regression line with a negative intercept on the Y-axis, and all permuted R^2^ and Q^2^ values were lower than those of the original model. This robustly demonstrates the model was not overfitted and the results are reliable (**Supplementary Figure 5A**). Similarly, for the LPS vs. LPS-PcDNs comparison, the OPLS-DA model also successfully distinguished the two groups (R^2^Y=0.967, Q^2^=0.951), and its validity was again confirmed by the permutation test. Variable Importance in Projection (VIP) plot analysis showed a significant number of metabolites with VIP scores >1.0 in both comparisons (312 for CTRL vs. LPS; 285 for LPS vs. LPS-PcDNs) (**Supplementary Figure 5B**). These metabolites with high VIP values contributed most to the inter-group separation and are potential biomarkers. The OPLS-DA results are highly consistent with the overall trends revealed by the aforementioned PCA. However, by employing a supervised method, they further amplified and quantified the inter-group differences. This demonstrates that LPS stimulation induced global and drastic reprogramming of the macrophage metabolic network, while intervention with P. cuspidatum-derived nanovesicles (PcDNs) reversed this pathological metabolic state, shifting the overall cellular metabolic profile back toward the normal physiological condition. This provides strong multivariate statistical evidence at a supervised level for the profound intervention effect of PcDNs on LPS-induced acute inflammation, laying a solid statistical foundation for the subsequent precise screening of key differential metabolites and in-depth investigation of their molecular mechanisms.

Principal component analysis (PCA) and hierarchical clustering (Heatmap) revealed distinct metabolic profiles among the Control, LPS, and LPS+PcDNs groups. The LPS challenge induced a profound metabolic shift, which was reversed by PcDNs treatment, driving the metabolic fingerprint back toward a homeostatic state (**Figures 5B, C**). Pathway enrichment analysis based on the KEGG database identified several key metabolic axes targeted by PcDNs, with glycerophospholipid metabolism and amino acid metabolism being the most impacted (**Figures 5D**, **6A**). Given the critical role of lipids in membrane integrity and inflammatory signaling, the modulation of glycerophospholipid metabolism highlights the capacity of PcDNs to repair cellular damage.

**Figure 6:**
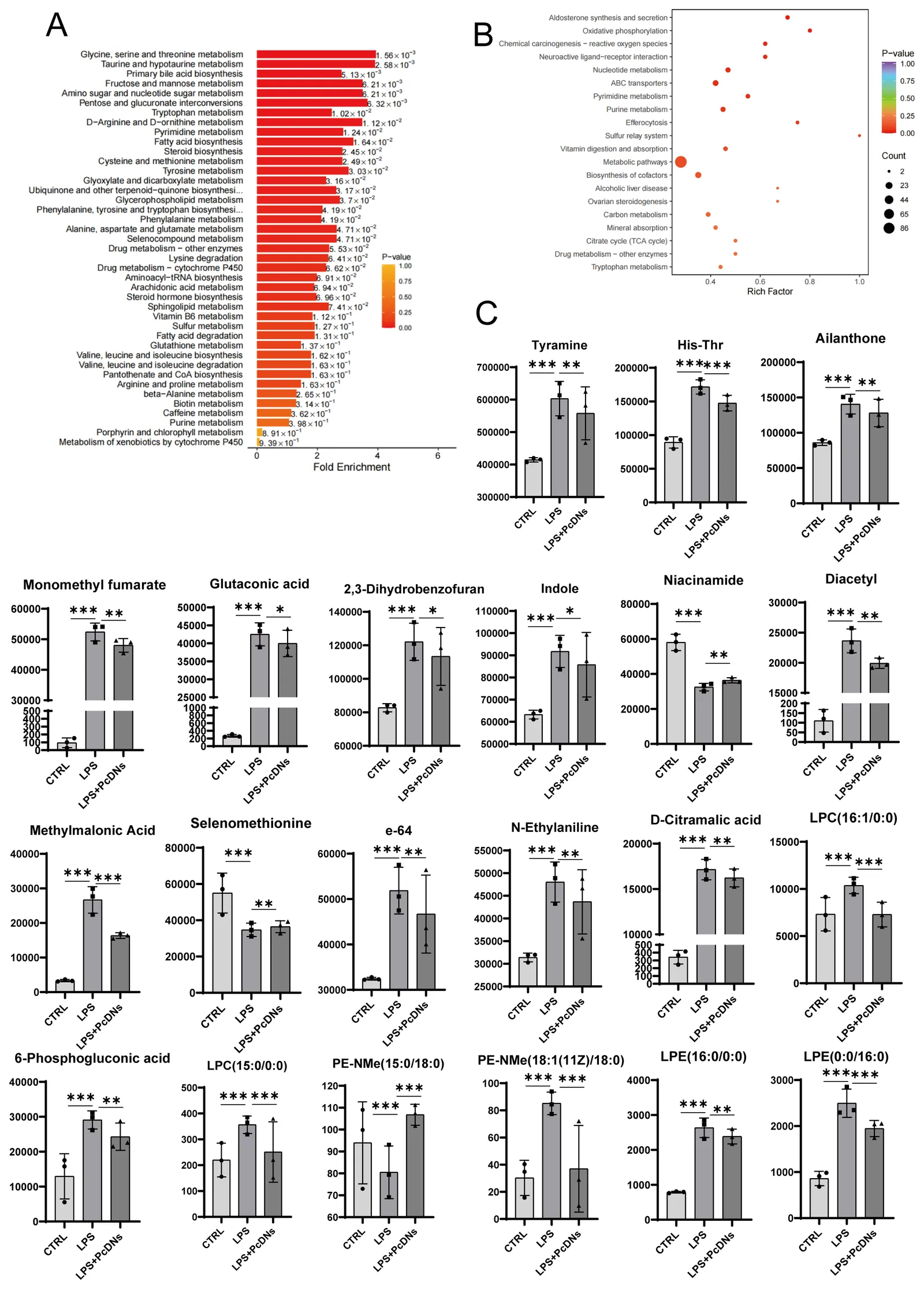
Pathway enrichment analysis and quantitative validation of key differential metabolites. **(A)** Heatmap of metabolic pathway enrichment. **(B)** Bubble plot for the identification of key differential metabolites. **(C)** Quantitative validation of representative differential metabolites across the Control (CTRL), LPS-stimulated, and LPS+PcDNs groups. Data are presented as mean ± SD (n=3). *P < 0.05, **P < 0.01, ***P < 0.001.

To pinpoint the specific drivers of this metabolic reprogramming, we analyzed the differentially expressed metabolites (DEMs) across the groups (**Figure 6B**). The top 20 pathways ranked by P-value were selected and displayed in ascending order to demonstrate that multiple key metabolic pathways were significantly enriched. Among them, the enrichment of “Metabolic pathways” was the most significant, with a high enrichment factor and a total of 86 differential metabolites enriched in this pathway. This may indicate that the intervention of PcDNs has a wide and profound impact on the basic material and energy metabolism network of cells. “Pyrimidine metabolism” and “Purine metabolism” also showed high enrichment significance. These two pathways are the core of nucleotide synthesis, and their disorders are closely related to cellular energy imbalance and abnormal proliferation signals. “Neuroactive ligand-receptor interaction” was also significantly enriched. This pathway involves extensive cell signal transduction. In addition, “Chemical carcinogenesis - reactive oxygen species”, which is closely related to oxidative stress, was also enriched, indicating that PcDNs may alleviate oxidative damage caused by inflammation by regulating ROS-related metabolism. Quantitative validation confirmed that PcDNs treatment restored the levels of several bioactive metabolites that were dysregulated by LPS. We observed a marked increase in the levels of monomethyl fumarate (an anti-inflammatory metabolic intermediate) and indole (a key ligand for the aryl hydrocarbon receptor that promotes immune tolerance) following PcDNs administration (**Figure 6C**). Furthermore, specific species of phosphatidylethanolamines (PEs) and lysophosphatidylcholines (LPCs), particularly within the PE-NMe class, were modulated (**Figure 6C**). These results suggest that PcDNs do not simply act as a drug carrier, but also function as a biological modulator that restores metabolic homeostasis and suppresses inflammation through the orchestration of endogenous metabolic pathways.

### 3.5 Dual prophylactic and therapeutic anti-inflammatory potency of PcDNs@Dex *in vitro*

To further evaluate the clinical translational potential and versatility of the PcDNs@Dex platform, we investigated its efficacy under two distinct administration regimens: therapeutic and preventive (**Figure 7A**).

**Figure 7:**
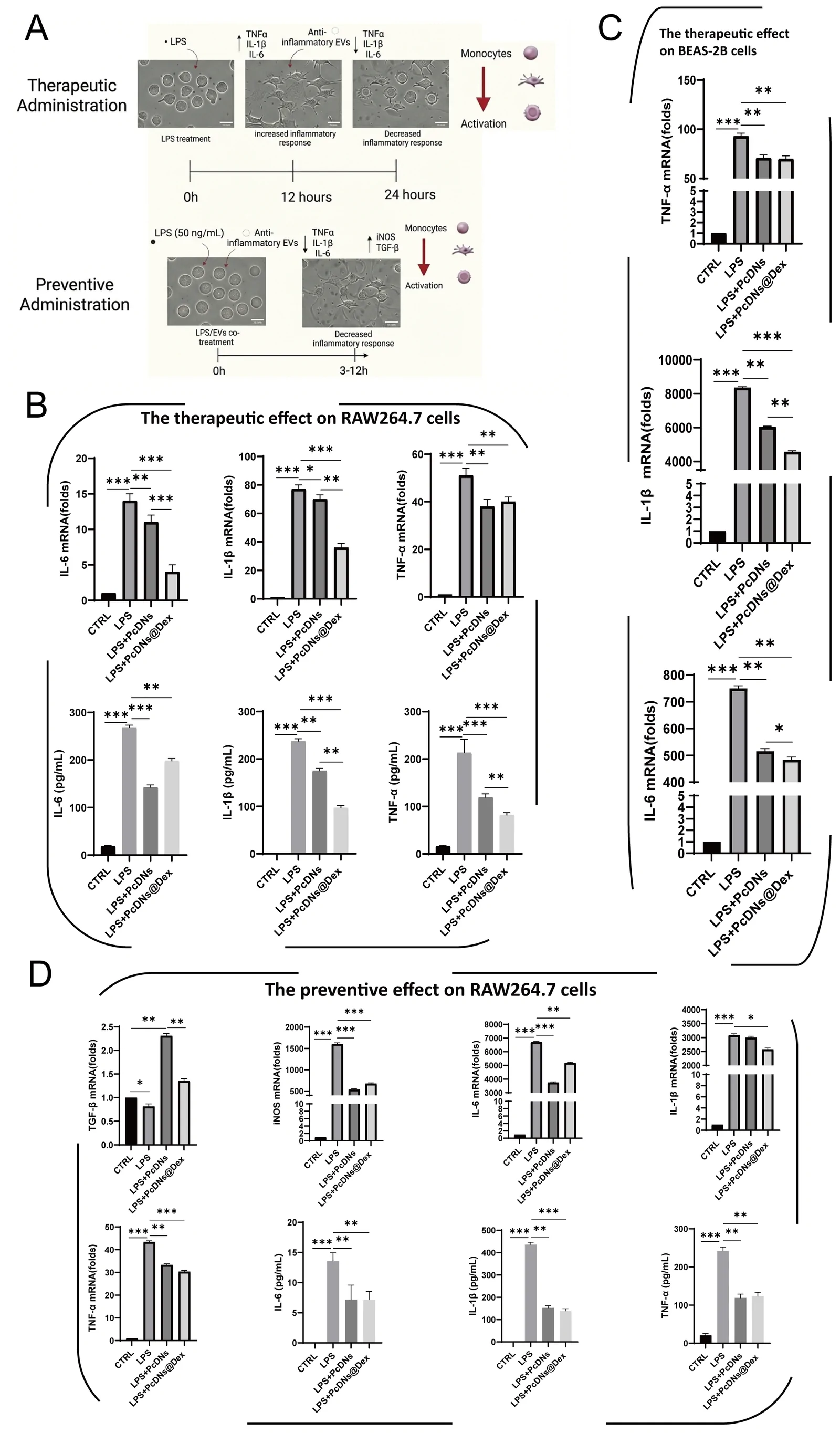
Therapeutic and preventive anti-inflammatory activities of PcDNs@Dex *in vitro*. **(A)** Schematic illustration of therapeutic and preventive administration regimens. **(B)** Therapeutic efficacy of PcDNs@Dex in LPS-induced RAW264.7 macrophages. mRNA expression and protein secretion of TNF-α and IL-6 were measured by qRT-PCR and ELISA, respectively. **(C)** Therapeutic efficacy of PcDNs@Dex in LPS-induced BEAS-2B bronchial epithelial cells. mRNA expression of IL-6 was analyzed by qRT-PCR. **(D)** Preventive effects of PcDNs@Dex in LPS-induced RAW264.7 macrophages. mRNA expression and protein secretion of TNF-α and IL-6 were evaluated by qRT-PCR and ELISA. Data are presented as mean ± SD (n=3). Statistical significance was determined by one-way ANOVA followed by appropriate post-hoc test. *P < 0.05, P < 0.01,*P < 0.001 compared with the LPS group.

In the therapeutic model, RAW264.7 macrophages were first primed with LPS to establish a robust inflammatory state before the introduction of PcDNs@Dex. Quantitative RT-PCR and ELISA results demonstrated that PcDNs@Dex reversed the established inflammatory response, characterized by a marked reduction in the mRNA expression and protein secretion of key pro-inflammatory cytokines, including TNF-α and Il-6 (**Figure 7B**). Similar therapeutic benefits were observed in BEAS-2B bronchial epithelial cells, where the LPS-induced upregulation of Il-6 was effectively attenuated (**Figure 7C**). These data suggest that PcDNs@Dex can potently resolve active inflammation by suppressing the hyperactivation of both immune and structural lung cells.

Beyond its therapeutic reversal capacity, we sought to determine whether PcDNs@Dex could confer prophylactic protection against inflammatory insults. In the preventive regimen, cells were pre-conditioned with PcDNs@Dex prior to LPS challenge, leading to a blunting of the subsequent inflammatory surge as evidenced by diminished levels of TNF-α and IL-6 (**Figure 7D**). This prophylactic effect indicates that PcDNs and PcDNs@Dex might stabilize the cellular microenvironment or program the cells into a state of “endotoxin tolerance.” Collectively, these findings highlight the dual-functional role of PcDNs and PcDNs@Dex, demonstrating its capability to both treat acute inflammatory episodes and provide a protective shield against potential inflammatory challenges.

### 3.6 Nebulized PcDNs@Dex attenuates acute lung injury and ensures systemic safety *in vivo*

To establish a stable and reliable mouse model of acute pneumonia, we systematically screened the modeling concentration via intraperitoneal injection of lipopolysaccharide (LPS). Experimental groups included a control group (Ctrl) and three dose groups (3 mg/kg, 7 mg/kg, and 15 mg/kg). A comprehensive assessment was performed by quantifying the mRNA expression levels of key pro-inflammatory cytokines (IL-6, IL-1β, and TNF-α) in lung tissue using qPCR, combined with histopathological evaluation of lung tissue sections (H&E staining) (**Supplementary Figure 6**). Based on the expression intensity of the inflammatory factors and the degree of tissue pathological damage, 7 mg/kg intraperitoneal LPS was determined to be the optimal dose for establishing the acute pneumonia model, and it was subsequently used for therapeutic research.

The therapeutic efficacy of nebulized PcDNs@Dex was first demonstrated by its ability to preserve lung structure and function (**Figure 8A**). Compared to the control group, LPS-challenged mice exhibited significant body weight loss (**Figure 8B**) and severe histopathological lung injury, characterized by alveolar wall thickening, inflammatory cell infiltration, and architectural disruption (**Figure 8F**). Treatment with nebulized PcDNs@Dex markedly ameliorated these pathological changes. Quantitative morphometric analysis confirmed that PcDNs@Dex reduced alveolar septal thickening, increased the number of alveoli per field, and restored the mean linear intercept (MLI) towards normal levels, indicating effective protection of the alveolar architecture (**Figure 8G**).

**Figure 8:**
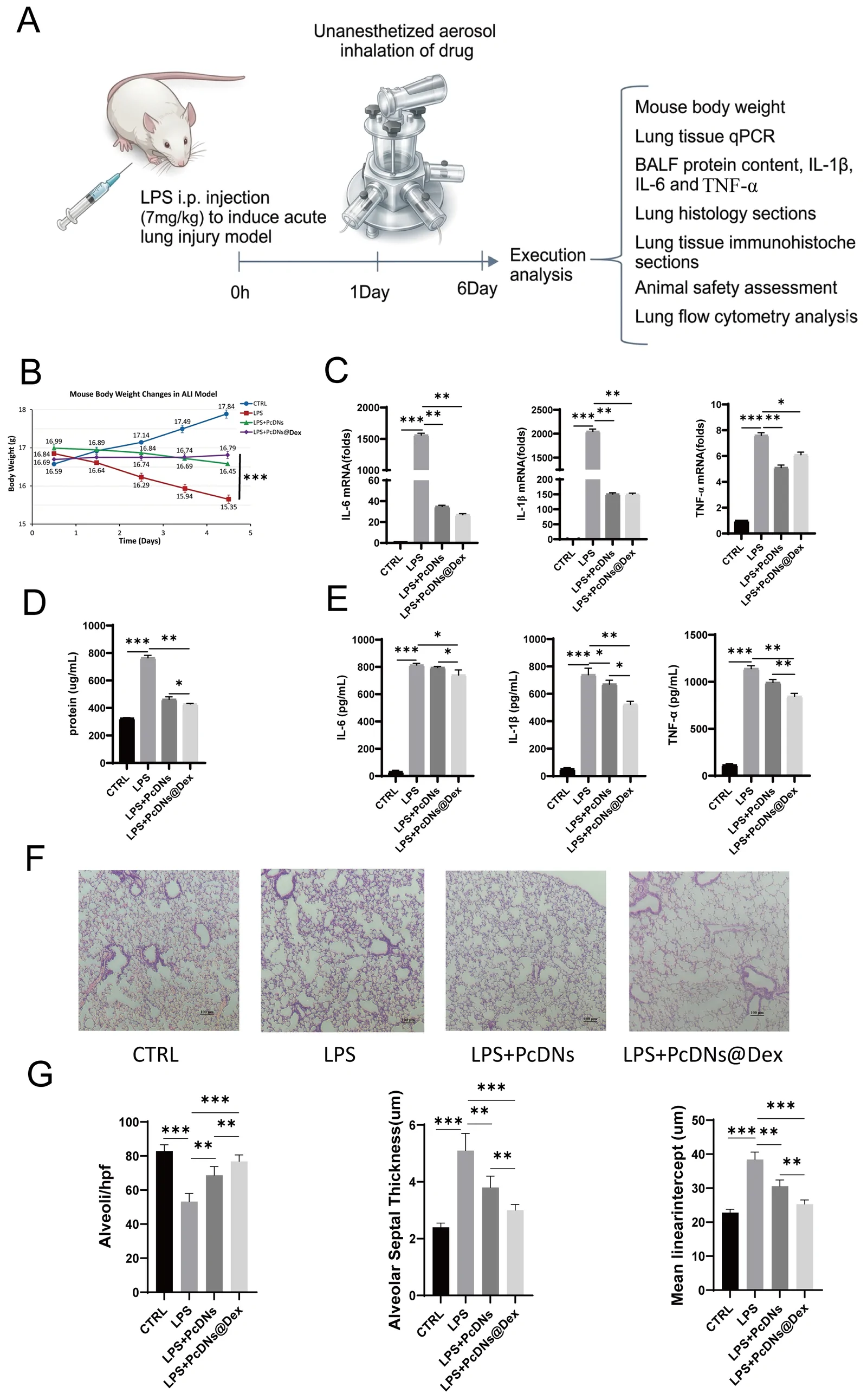
Safety and therapeutic efficacy of nebulized PcDNs@Dex in an LPS-induced mouse model of acute lung injury. **(A)** Schematic illustration of the experimental paradigm. **(B)** Dynamic body weight changes. **(C)** Transcriptional levels of inflammatory cytokines (IL-6, IL-1β, TNF-α) in lung tissues measured by qPCR. **(D)** Total protein concentration in bronchoalveolar lavage fluid (BALF). **(E)** Protein levels of IL-6, IL-1β, and TNF-α in BALF measured by ELISA. **(F)** Representative H&E-stained lung tissue sections (Scale bar = 100 µm). **(G)** Quantitative histopathological assessment of lung injury, including number of alveoli per high-power field, Alveolar Septal Thickness and mean linear intercept. Data are presented as mean ± SD (n=6). Statistical significance was determined by one-way ANOVA followed by appropriate post-hoc test. *P < 0.05, **P < 0.01, ***P < 0.001.

Consistent with the structural improvement, PcDNs@Dex potently suppressed the inflammatory “cytokine storm” at both the transcriptional and protein levels. In lung tissues, PcDNs@Dex downregulated the LPS-induced mRNA expression of Il-6, Il-1β, and TNF-α (**Figure 8C**). This suppression was confirmed at the protein level. Treatment with PcDNs and PcDNs@Dex reduced the concentrations of IL-6, IL-1β, and TNF-α in bronchoalveolar lavage fluid (BALF) (**Figure 8E**). Furthermore, the total protein concentration in BALF was significantly lower in the PcDNs and PcDNs@Dex group compared to the LPS model group (**Figure 8D**), underscoring the platform’s role in restoring vascular integrity.

These results were consistent with immunohistochemical (IHC) staining and qPCR analysis of lung parenchyma, which showed a drastic downregulation of pro-inflammatory mediators across the lung tissue (**Figure 9A**). Mechanistically, we investigated whether the *in vivo* efficacy was associated with immune cell reprogramming. Flow cytometric analysis of lung dissociates revealed that the PcDNs@Dex treatment modulated the macrophage landscape (**Figures 9B, C**). Specifically, there was a notable increase in the percentage of CD206+ (M2-like) anti-inflammatory macrophages compared to the LPS model group, suggesting that the nebulized nanovesicles promote a transition from a destructive inflammatory phase to a reparative phase within the lung microenvironment.

**Figure 9:**
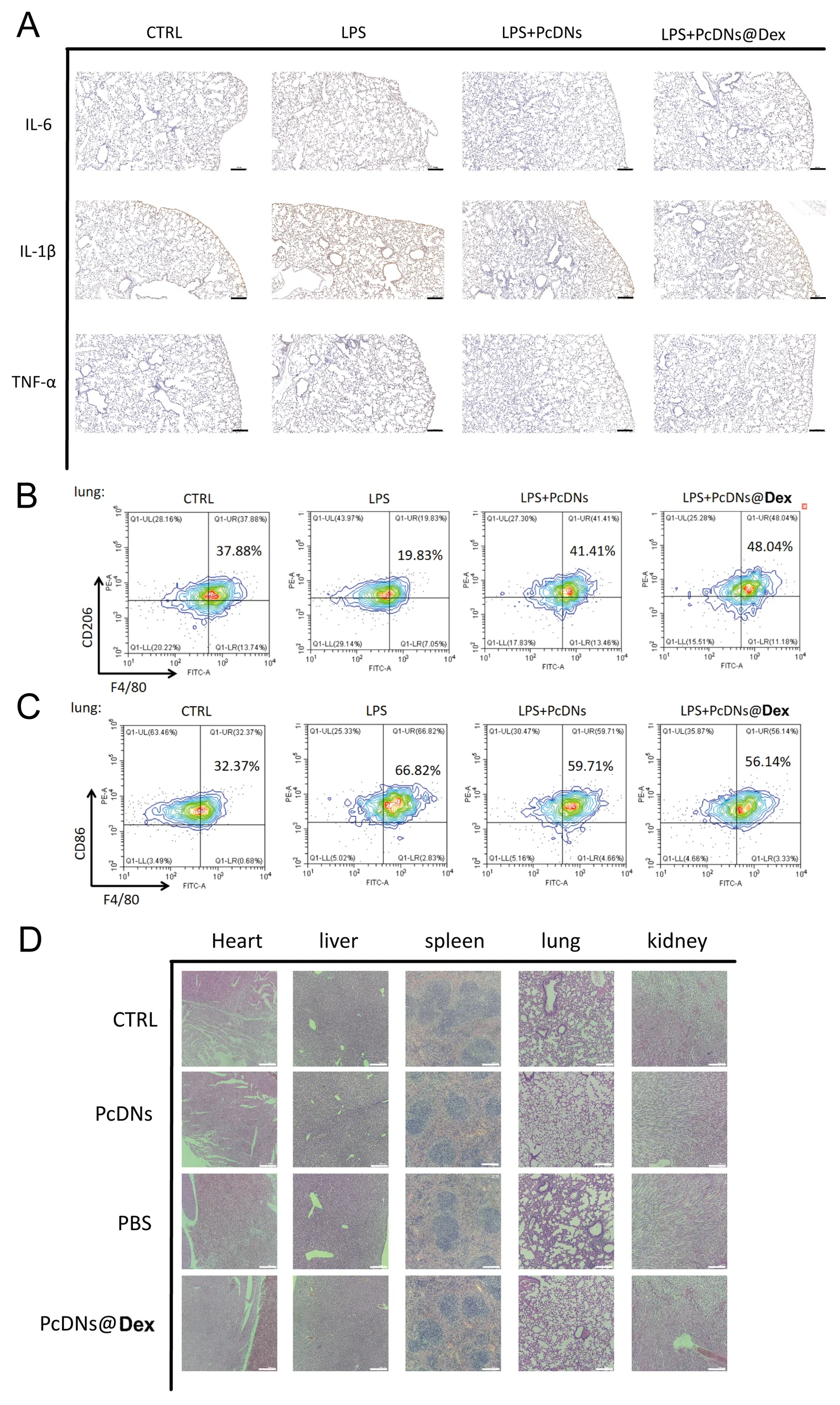
PcDNs@Dex suppresses pulmonary inflammation, modulates macrophage subpopulations, and exhibits a favorable systemic safety profile. **(A)** Representative immunohistochemical (IHC) images of IL-6, IL-1β, and TNF-α in lung tissues (Scale bar = 100 μm). **(B, C)** Flow cytometric analysis of macrophage subpopulations in lung dissociates. **(B)** Percentage of CD206^+^ (M2-like) macrophages. **(C)** Percentage of CD86^+^ (M1-like) macrophages. **(D)** Representative H&E-stained sections of heart, liver, spleen, lung, and kidney. Data are presented as mean ± SD (n=3). Statistical significance was determined by one-way ANOVA followed by appropriate post-hoc test.

Finally, we evaluated the systemic safety of the nebulized platform, which is a critical parameter for clinical application. H&E staining of major metabolic and vital organs, including the heart, liver, spleen, and kidney, revealed no detectable pathological changes or organic damage across all treatment groups (**Figure 9D**). These findings, coupled with the localized nature of the inhalation therapy, demonstrate that PcDNs@Dex possesses an safety profile and biocompatibility, positioning it as a promising candidate for the targeted treatment of inflammatory lung diseases.

## 4 Discussion

In this study, we successfully engineered a bio-inspired nanoplatform utilizing exosome-like nanovesicles derived from the medicinal herb Polygonum cuspidatum(PcDNs) for the targeted pulmonary delivery of dexamethasone. We demonstrated that PcDNs@Dex, optimized via electroporation, maintains exceptional physicochemical stability and structural integrity after nebulization, a critical prerequisite for effective inhalation therapy. Notably, the nanovesicles retained their intrinsic anti-inflammatory bioactivity post-nebulization, functioning not merely as passive carriers but as active participants in immunometabolic reprogramming. This PcDNs-based inhalation system overcomes a key bottleneck in current pharmacotherapy—namely, the inability to achieve sustained, high local drug concentrations at the primary site of lung injury while minimizing systemic off-target effects. By coupling efficient pulmonary delivery with inherent therapeutic activity, our platform represents a convergent strategy that addresses both the pharmacokinetic and mechanistic limitations of conventional systemic corticosteroid therapy for inflammatory lung diseases (23–28).

Crucially, PcDNs transcend the paradigm of a passive “delivery vehicle”. A key advancement of this work is recognizing and leveraging their intrinsic biological activity. Plant-derived vesicles are not mere empty shells; they encapsulate a rich cargo of bioactive molecules from their source plant, including proteins, lipids, miRNAs, and secondary metabolites (29–32). In the case of P. cuspidatum, this cargo inherently includes compounds like resveratrol and polydatin, known for their anti-inflammatory and antioxidant properties (33, 34). Our data provide strong evidence for this “dual-function” hypothesis: PcDNs alone exhibited significant anti-inflammatory effects *in vitro*, and the observed synergistic efficacy of PcDNs@Dex cannot be explained by simple additive effects. This positions PcDNs as a bioactive nano-platform—a synergistic combination of a sophisticated drug delivery system and a source of natural therapeutic compounds (31).

Despite these therapeutic merits, a key limitation constraining the clinical translation of plant-derived nanovesicles is the bottleneck of large-scale, industrial-grade yield amplification. Conventional ultracentrifugation-based isolation workflows, as employed in this study, generally suffer from low recovery efficiency, high processing cost and poor scalability, and are difficult to adapt to the batch-to-batch consistency and yield requirements of pharmaceutical manufacturing. Meanwhile, the absence of harmonized quality control standards for vesicle purity, structural integrity and bioactivity retention further adds to the translational barriers (35–37). Looking forward, future research should prioritize the development of high-efficiency, low-cost continuous purification technologies and standardized quality evaluation systems to break through the production scale-up bottleneck of PcDNs and facilitate their bench-to-bedside translation.

The superior anti-inflammatory efficacy of PcDNs@Dex can be attributed to its profound reprogramming of the pulmonary immune microenvironment, with macrophage polarization serving as a central node. In sepsis-induced ALI, alveolar macrophages are primed towards a pro-inflammatory M1 phenotype, a key driver of tissue damage and alveolar-capillary barrier disruption (38, 39). Our findings that PcDNs@Dex shifted the M1/M2 balance towards the reparative M2 phenotype are of paramount therapeutic significance. M2 macrophages are not merely anti-inflammatory; they actively secrete factors like IL-10 and TGF-β that promote tissue repair, dampen neutrophil infiltration, and facilitate the restoration of the epithelial barrier (40, 41). This shift from a destructive to a regenerative immune milieu is crucial for resolving ALI and preventing progression to fibrosis (39, 42). Importantly, the robust prophylactic efficacy of PcDNs@Dex observed in **Figure 7** further expands its clinical application scope, demonstrating promising potential for the prevention of high-risk ALI. Clinically, perioperative inflammatory stress and pathogenic infection are two predominant triggers of acute lung injury, which often lead to abrupt pulmonary immune dysregulation and poor patient prognosis; targeted prophylactic intervention before inflammatory cascade activation is therefore critical for reducing ALI incidence and mortality (43–45). As a novel inhalation therapeutic agent, PcDNs@Dex can exert preemptive immunomodulatory effects to inhibit early M1-type macrophage activation and block the initiation of pulmonary inflammatory injury, which aligns well with the clinical demand for perioperative and infection-associated ALI prevention. Additionally, the combined regimen inherits the reliable anti-inflammatory advantages of dexamethasone while overcoming its limitations of systemic side effects and single-target regulation, providing an optimized prophylactic strategy for glucocorticoid-based ALI intervention (46, 47).

Our untargeted metabolomics analysis elevates the mechanistic understanding of PcDNs@Dex from the level of immune cell modulation to the fundamental realm of cellular metabolism. The most significant finding was the profound normalization of glycerophospholipid metabolism, a pathway that emerged as the core metabolic axis perturbed by LPS and rescued by PcDNs intervention. This is not merely an associative observation; glycerophospholipids are central to lung homeostasis. They are essential structural components of both cellular membranes and pulmonary surfactant, the lipoprotein complex critical for preventing alveolar collapse and serving as a first line of innate immune defense (48). Dysregulated glycerophospholipid metabolism, as seen in our LPS model, compromises membrane integrity in alveolar epithelial and endothelial cells, directly contributing to barrier dysfunction and edema (49). By restoring this metabolic pathway, PcDNs@Dex likely reinforces cellular membrane architecture and surfactant stability, thereby fortifying the physical barriers against inflammation and injury. Our study positions PcDNs as a bioactive agent capable of similar metabolic “repair,” moving beyond symptom suppression to address a root cause of tissue vulnerability.

## 5 Conclusion

In summary, this study establishes a dual-functional nanoplatform​that integrates drug delivery with intrinsic bioactivity, by engineering Polygonum cuspidatum-derived nanovesicles loaded with dexamethasone (PcDNs@Dex) (**Figure 10**). Beyond acting as a simple carrier, PcDNs function as active immunometabolic modulators, coupling efficient pulmonary delivery of dexamethasone with inherent macrophage reprogramming and glycerophospholipid metabolic restoration.

**Figure 10:**
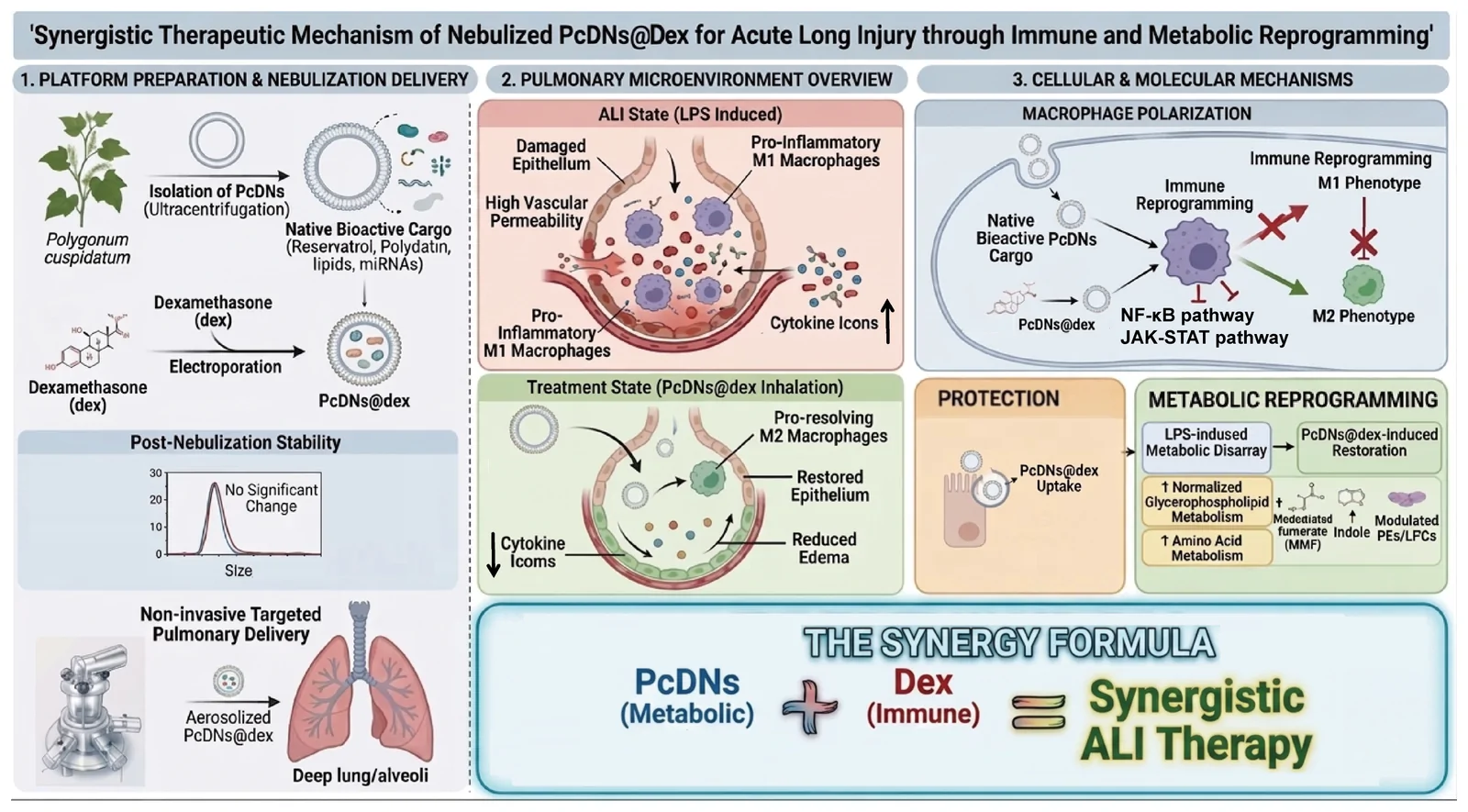
Schematic diagram of the application of PcDNs@Dex in acute lung injury.

This work represents a paradigm shift in inhalation therapy: unlike conventional nebulized formulations that rely solely on passive drug deposition, our platform leverages bioactive plant-derived nanovesicles that survive the mechanical stress of nebulization and actively reshape the diseased lung microenvironment. The demonstration of both prophylactic and therapeutic efficacy, alongside excellent systemic safety, validates this approach as a clinically viable alternative to systemic corticosteroid therapy.

Mechanistically, the platform operates through a synergistic dual axis—immune modulation (M1→M2 macrophage polarization via NF-κB/JAK-STAT inhibition) and metabolic repair (restoration of glycerophospholipid homeostasis)—distinguishing it from single-target interventions. This positions PcDNs@Dex as a prototype of plant-derived advanced therapy medicinal products (ATMPs)​ for inflammatory lung diseases.

By bridging natural product chemistry, nanomedicine, and immunometabolism, this work provides a blueprint for developing next-generation, sustainable, and multifunctional inhalable therapeutics, paving the way for clinical translation of plant-derived ATMPs in respiratory medicine.

## Data Availability

The datasets presented in this study can be found in online repositories. The names of the repository/repositories and accession number(s) can be found in the article/**Supplementary Material**.
