# Supplementary material for: Nebulized Polygonum cuspidatum-derived nanovesicles loaded with dexamethasone synergistically ameliorate acute lung injury via metabolic-immune reprogramming
Source: Front Immunol. 2026 Sep 22;17:1872209. doi: 10.3389/fimmu.2026.1872209 (PMC13640951; doi:10.3389/fimmu.2026.1872209)
Supplement: Supplementary file 1 [file Table1.docx]

Supplementary Material

**Supplemental Fig. 2.** Establishment and optimization of lipopolysaccharide-induced cell inflammation models (n=3). (A) Effects of LPS modeling concentration and time on the mRNA expression of inflammatory factors in RAW264.7 cells. (B) Effects of LPS modeling concentration and time on the mRNA expression of inflammatory factors in BEAS-2B cells. *P < 0.05, **P < 0.01. ns, not significant.

**Supplemental Fig. 3.** Quality assessment of the untargeted metabolomics data. (A) Superimposed total ion chromatogram (TIC) of QC sample mass spectrometry detection. The results show that the curves of total ion flow for metabolite detection have high overlap, that is, both retention time and peak intensity are consistent, indicating that the signal stability of mass spectrometry for the same sample at different times is good. The high stability of the instrument provides an important guarantee for the repeatability and reliability of the data. (B) Pearson correlation heatmap between QC samples. The diagonal squares represent the names of QC samples; the squares in the lower left corner of the diagonal are the corresponding QC sample correlation scatter plots, with the horizontal and vertical coordinates representing the metabolite content (log-transformed), and each point in the figure represents a metabolite; the squares in the upper right corner of the diagonal are the Pearson correlation coefficients of the corresponding QC samples.(C) Distribution of coefficient of variation (CV) of metabolites. The horizontal axis represents the CV value, and the vertical axis represents the proportion of substances with a CV value less than the corresponding value to the total number of substances. Different colors represent different group samples, and QC represents QC samples. The two reference lines perpendicular to the X-axis correspond to CV values of 0.3 and 0.5, and the two reference lines parallel to the X-axis correspond to 75% and 85% of the total number of substances.

B

A

**Supplemental Fig. 4.** Unsupervised principal component analysis of untargeted metabolomics data. (A) 2D PCA score plot. (B) Statistical process control chart for the PC1 values of QC samples.

**Supplemental Fig. 5.** OPLS-DA-based analysis of inter-group metabolic profile differences: Supervised OPLS-DA was performed for the CTRL vs LPS (A) and LPS vs LPS-PcDNs (B) comparisons to quantify inter-group metabolic differences.

**Supplemental Fig. 6.** Dose screening for establishing a mouse model of acute pneumonia via intraperitoneal LPS injection. (A) The mRNA contents of IL-6, IL-1β and TNF-α in the lung tissues of the four different-dose modeling groups were compared. *P < 0.05, # indicated significant differences compared with the control group, and p value < 0.05. (B) The HE-stained sections of the lung tissues from the four different-dose modeling groups.
